# Sex differences in the association between preexisting comorbidities and COVID-19-related symptoms during the COVID-19 pandemic in the Dominican Republic

**DOI:** 10.3389/fpubh.2025.1536627

**Published:** 2025-03-18

**Authors:** Shu-Hui Wen, Beatrice Chakanika, Nelson Martínez Rodríguez, Katherine Victorio Suberví, Julia Pérez Rodríguez, Lih-Ming Yiin, Chia-Jung Hsieh

**Affiliations:** ^1^Department of Public Health, College of Medicine, Tzu Chi University, Hualien, Taiwan; ^2^Health Research Directorate, Ministry of Public Health, Santo Domingo, Dominican Republic

**Keywords:** asthma, chronic lung disease, hypertension, diabetes mellitus, COVID-19-related symptoms, sex difference

## Abstract

**Background:**

Sex-based differences in the impact of comorbidities on coronavirus disease 2019 (COVID-19) related symptoms remain underexplored due to the predominance of sex-aggregated data. We aimed to examine sex differences in the associations between preexisting comorbidities and COVID-19-related symptoms during the COVID-19 pandemic in the Dominican Republic.

**Methods:**

We conducted a cross-sectional study using a questionnaire survey in the Dominican Republic between September 2021 and December 2021. Data on demographic factors, preexisting comorbidities, and self-reported COVID-19-related symptoms were collected. A multiple logistic regression model was used to separately identify associations between preexisting comorbidities and COVID-19-related symptoms in males and females.

**Results:**

We included a total of 3,308 eligible individuals. Approximately 25% of the participants had preexisting comorbidities, and 31% of the participants experienced COVID-19-related symptoms. Multiple logistic regression analyses revealed that asthma (OR = 2.15, 95% CI = 1.20–3.85, *p* = 0.01) was associated with the presence of COVID-19-related symptoms in males. For females, chronic lung disease (OR = 5.39, 95% CI = 1.52–19.18, *p* = 0.009), hypertension (OR = 1.33, 95% CI = 1.00–1.77, *p* = 0.047) and diabetes mellitus (OR = 1.70, 95% CI = 1.07–2.71, *p* = 0.025) were correlated with COVID-19-related symptoms.

**Conclusion:**

Our study findings observed sex-differences in the associations between preexisting comorbidities and COVID-19-related symptoms. Specifically, we observed that male individuals with asthma and females with chronic lung disease, hypertension, and diabetes mellitus had a greater likelihood of experiencing COVID-19-related symptoms. Future studies are needed to confirm the mechanism underlying these sex differences.

## 1 Introduction

Since 2020, the coronavirus disease 2019 (COVID-19) pandemic has spread worldwide, including to the Dominican Republic. It has been reported that as of February 14, 2024, there were 675,890 and 4,384 COVID-19-related cases and deaths, respectively, in the Dominican Republic (1). The Dominican Republic is an upper-middle-income country (2), with the most of its population identifying as mixed ethnicity. The sex ratio of males versus females is 1.02 (3). We aimed to utilize this opportunity to investigate sex differences in COVID-19-related symptoms within the context of the local epidemiological characteristics and population structure. This study provides valuable epidemiological data that can serve as a crucial reference for future public health decision-making and disease prevention strategies.

The symptoms of COVID-19 determine the protocols for isolation and testing and influence the severity of the illness and the necessity for hospitalization (4, 5). A broad spectrum of symptoms is linked to COVID-19, ranging from mild discomfort to severe illness (6). Common symptoms include cough, shortness of breath, difficulty breathing, fatigue, muscle or body aches, headache, loss of taste or smell, sore throat, congestion or runny nose, nausea or vomiting, and diarrhea (7). Emerging evidence from previous studies has shown that underlying chronic diseases are associated with COVID-19 symptoms (8–14). However, our understanding of sex differences in how comorbidities affect individuals differently is limited by the reliance on sex-aggregated analyses in the COVID-19 literature. Sex differences have been observed in COVID-19 infection rates, symptom presentations, severity, and mortality outcomes (15–18). However, few studies have investigated sex differences in association between comorbidities and COVID-19 symptoms. It is known that differences between males and females exist in the genetics, immune response, sex hormones, and socioeconomic and behavioral factors that are relevant to health and disease (19, 20). Filling this gap would provide valuable information for sex-specific clinical management and public health strategies. A better understanding of these differences is the key for physicians to diagnose effectively and determine appropriate management strategies.

Over 50% of COVID-19 patients have at least one comorbidity, with hypertension (16.6%) being the most common, followed by diabetes (7.5%), hepatitis B (3.1%), and heart disease (1.5%) (21). Previous reports have shown that more severe symptoms are associated with comorbidities such as hypertension, diabetes, cancer, obesity, chronic lung disease, chronic liver disease, and cardiovascular disease (13, 22, 23). Additionally, the prevalence of comorbidities such as hypertension and diabetes differed by sex in COVID-19 patients (24). These results indicate a potentially diverse impact of preexisting comorbidities on COVID-19 symptoms across sexes; however, further clarification is needed. The objective of this study was to separately examine the associations between preexisting comorbidities and COVID-19-related symptoms in male and female subjects. Particularly, we aimed to determine whether these associations exhibit sex-specific differences, utilizing epidemiological survey data from the Dominican Republic.

## 2 Materials and methods

### 2.1 Study participants

This cross-sectional study was an international project funded by the Tzu Chi Foundation. The research project collaborated with the Health Research Directorate of the Ministry of Public Health and Social Assistance of the Dominican Republic and the Department of Public Health at Tzu Chi University in Taiwan, employing announcements via public address to notify residents in targeted regions, including Santo Domingo, Santiago, Espaillat, and Bahoruco in the Dominican Republic. Educational materials for the importance of rapid testing and vaccination for COVID-19 were distributed to the public. The purpose was to encourage participants to engage in the study and undergo the COVID-19 IgM/IgG antibody test. Participants were invited to participate in this study based on their willingness to participate before the COVID-19 IgM/IgG antibody test. Subsequently, each participant enrolled in this study provided informed consent. Sufficient explanations of the content from experienced staff to the participants facilitated obtaining questionnaire responses. The process of obtaining responses to the questionnaires was facilitated by sufficient explanations of the content from experienced staff to the participants. Residents in the selected areas of the Dominican Republic were invited to participate in the study. Invitations were extended through strategies such as public announcements and health promoters’ distribution of educational materials, encouraging individuals to undergo COVID-19 testing. The surveys and antibody tests were conveniently administered in parks and plazas across the selected provinces, facilitated by the coordination of the Provincial Health Directorates.

**Figure 1:**
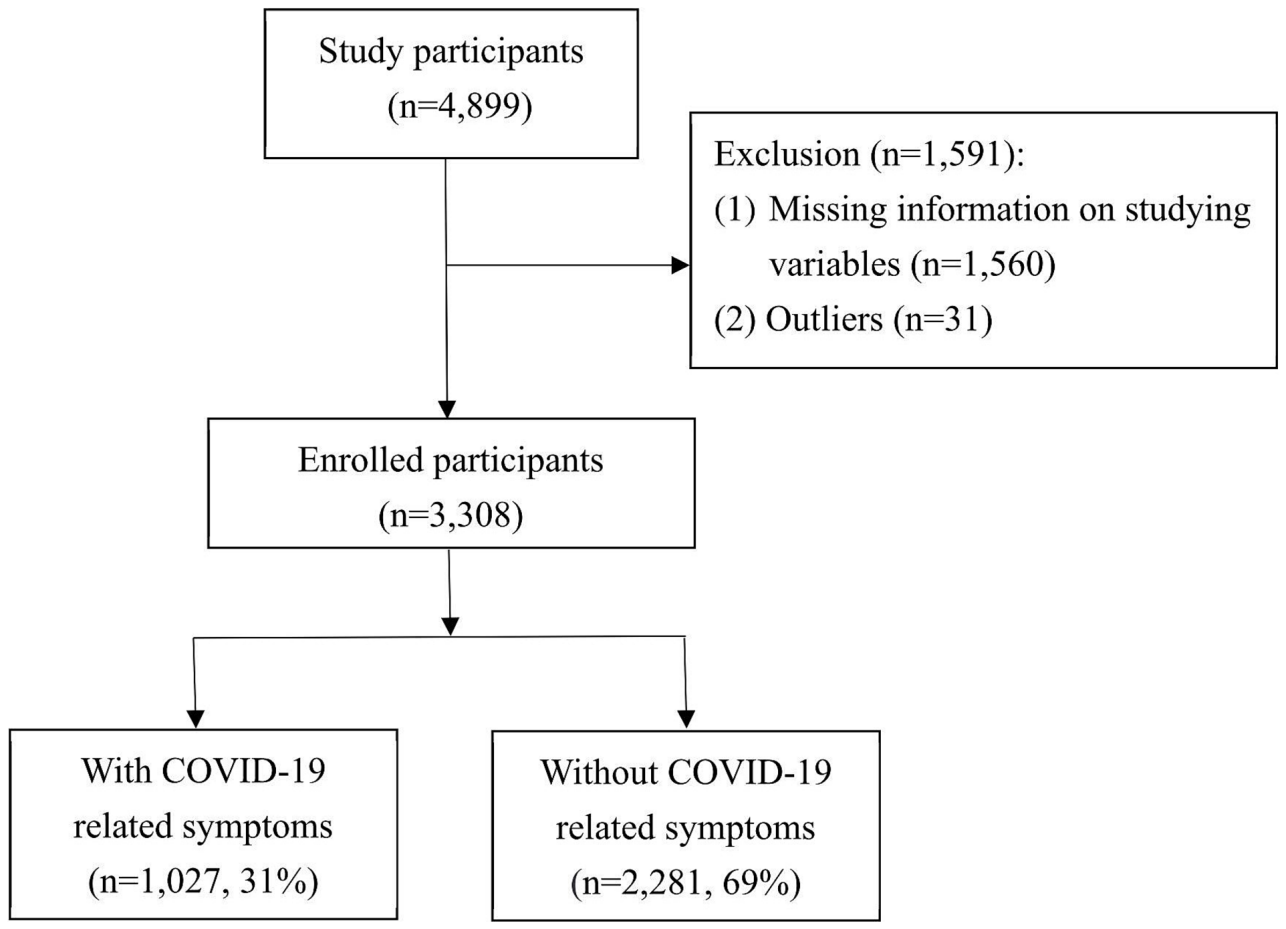
Flow diagram of participants included in this study.

The sample size was calculated using epiR package in R language. For a case–control study with pre-assumed 20% of individuals with at least one comorbidity in the control group (individuals without COVID-19 symptoms), the odds ratio of 1.36 (12), 95% confidence level, and 80% power, the minimum total sample size was estimated to be 1906. We then targeted the number of participants to meet the minimum requirement. We then targeted the number of participants to meet the minimum requirement. Data was collected among mentally and physically capable adults between September 2021 and December 2021. A total of 4,899 individuals were included, all of whom provided valid questionnaire responses. Among these participants, 1,591 were subsequently excluded due to missing information on study variables and outliers. Nevertheless, the limitation of medical resources and the inability of the participants to recall led to a high rate of missing data, with a lack of BMI data in approximately 54.5% (n = 851) of the missing data. Thus, 3,308 participants were included for subsequent data analysis (Figure 1).

Ethical approval was obtained from the National Council of Bioethics in Health of the Dominican Republic (CONABIOS 012–2021) and the research ethics committee of Tzu Chi Hospital, Taiwan (IRB109-268-B). Informed consent was obtained from each respondent.

### 2.2 Data collection

A self-reported structured questionnaire was designed for this epidemiological survey. The data included sociodemographic variables, preexisting comorbidities, and COVID-19-related symptoms. The sociodemographic variables included sex (male vs. female), age (years), height, weight, education level (uneducated and primary, junior and senior high school, and above university), marital status (unmarried, married, and divorced, and other categories including free union, separated, and widowed), race, and ethnic background (Hispanic, Black, White, and other categories including Asian/Pacific Islander, mixed race, other, and unknown), smoking status (yes or no), place of residence (rural village and urban area), household size (less than or more than 2 people in a house), and working in medical settings (yes or no). Additionally, body mass index (BMI) was calculated by dividing an individual’s weight in kilograms by their height in square meters.

Preexisting comorbidities included asthma, chronic lung disease, chronic obstructive pulmonary disease, hypertension, diabetes mellitus, coronary heart disease, chronic liver disease, chronic kidney disease, and autoimmune/rheumatic disease. These comorbidities were self-reported by participants and were not verified by medical professionals or laboratory testing due to limited medical resources. All comorbidities above were recorded as binary variables (1 = yes, 0 = no). More than one comorbidity is the coexistence of more than one chronic comorbidity in an individual. Furthermore, self-reported COVID-19-related symptoms were collected from participants who answered ‘Yes’ whether they ‘have experienced signs and symptoms of COVID-19 in the last month?’. COVID-19-related symptoms included respiratory symptoms (cough, shortness of breath, rhinorrhea, or sore throat), gastrointestinal symptoms (nausea, hypogeusia, or diarrhea), and other manifestations (fever, myalgia/muscle ache, fatigue, headache, confusion, loss of sense of smell, or loss of sense of taste). The primary outcome was the presence of any COVID-19-related symptoms. Participants with COVID-19-related symptoms were defined as those with at least one sign and symptom related to COVID-19, while those who did not have any self-reported signs or symptoms associated with COVID-19 in the last 1 month were defined as those without COVID-19-related symptoms. In addition, the COVID-19 vaccination status was also included in this study.

### 2.3 Statistical analysis

A comparative analysis was examined to examine the associations between preexisting comorbidities and self-reported COVID-19-related symptoms. The descriptive data are presented as the number (n) and percentage (%) for categorical variables and as the mean and standard deviation (SD) for continuous variables. The Chi-square test was used to compare categorical variables between participants with and without COVID-19-related symptoms, and a two-sample t-test was used to compare continuous variables between the two groups. Multiple logistic regression analysis assessed association between preexisting comorbidities and COVID-19-related symptoms. We chose logistic regression for this study because it is well-suited for analyzing binary outcomes and allows for the effectively adjusting of multiple confounding factors. Additionally, logistic regression provides easily interpretable odds ratios, which are suitable for understanding the strength of associations between independent variables and the outcome. Given that overall and specific comorbidities were analyzed, we implemented two models. Model 1 was performed to assess the presence of any comorbidity, while model 2 was performed to examine each specific comorbidity; both models were adjusted for demographic factors. The model was adjusted for the following demographic factors: age, BMI, smoking status, education level, marital status, race, place of residence, household size, and working in medical settings. The analysis was conducted separately for males and females. The Hosmer-Lemeshow test was performed to assess the validity of the logistic regression models. Adjusted odds ratio (OR) and 95% confidence intervals (CIs) are provided. To investigate the interactions between sex and comorbidities, as well as between educational level and marital status, we constructed models that included interaction terms for these factors. Additionally, we performed a sensitivity analysis with multiple imputations to handle missing data. A *p* value <0.05 was considered to indicate statistical significance. All analyses were performed using the SPSS Statistics version 21.0 (IBM, Armonk, NY, USA).

## 3 Results

Of the 3,308 participants included in the analysis, 31% reported COVID-19-related symptoms. Of these participants, 2,067 were females, accounting for 62.5%, and 1,241 were males, representing 37.5%. The distributions of demographic characteristics among the groups with and without COVID-19-related symptoms are presented in Table 1. Significant differences between the two groups were found in sex, age, education level, marital status, race, place of residence, household size, and COVID-19 vaccination status (*p* < 0.05). The regression model included these significant variables as covariates to control for potential confounding effects.

**Table 1:** The distribution of demographic characteristics among the study groups with and without COVID-19-related symptoms (*n* = 3,308).

Variables	COVID-19 related symptoms, *n* (%)		*p* value
	With (*n* = 1,027, 31%)	Without (*n* = 2,281, 69%)	
Sex			0.003*
Male	347 (33.8)	894 (39.2)	
Female	680 (66.2)	1,387 (60.8)	
Age (years); Mean ± SD	39.4 ± 13.5	40.5 ± 14.4	0.005*
BMI (kg/m^2^); Mean ± SD	26.4 ± 5.1	26.3 ± 5.2	0.896
Education level			<0.001*
Uneducated and primary school	153 (14.9)	420 (18.4)	
Junior and senior high school	378 (36.8)	950 (41.6)	
University level and above	496 (48.3)	911 (39.9)	
Smoking status			0.950
Yes	60 (5.8)	132 (5.8)	
No	967 (94.2)	2,149 (94.2)	
Marital status			0.027*
Single	403 (39.2)	970 (42.5)	
Married	316 (30.8)	600 (26.3)	
Divorced and others^a^	308 (30.0)	711 (31.2)	
Race/ethnic			<0.001*
Hispanic	742 (72.2)	1,713 (75.1)	
Black	83 (8.1)	258 (11.3)	
White	110 (10.7)	177 (7.8)	
Others^b^	92 (9.0)	133 (5.8)	
Place of residence			<0.001*
Rural village	518 (50.4)	1,397 (61.2)	
Urban area	509 (49.6)	884 (38.8)	
Household size			<0.001*
≤ 2	418 (40.7)	769 (33.7)	
> 2	609 (59.3)	1,512 (66.3)	
Working in medical settings			0.335
Yes	122 (11.9)	245 (10.7)	
No	905 (88.1)	2,036 (89.3)	
Vaccinated for COVID-19			0.001
Yes	872 (85.1)	1820 (80.0)	
No	153 (14.9)	455 (20.0)	

^a^Indicates free union, separated, and widowed; ^b^Indicates Asian/Pacific Islander, mixed race, other, and unknown; *Indicates significance (*p* value < 0.05); SD represents standard deviation.

**Figure 2:**
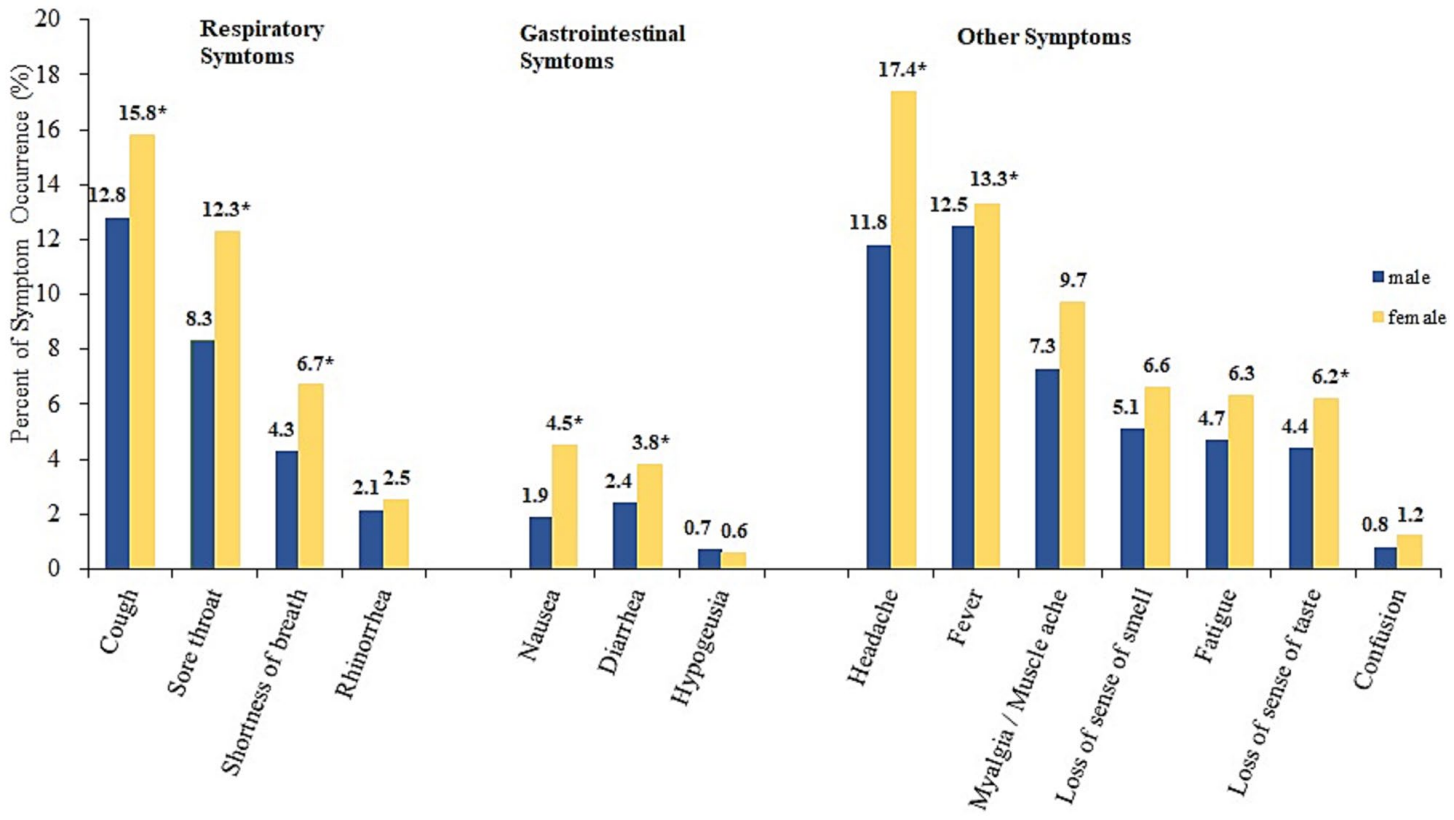
Percent of COVID-19-related symptoms stratified by sex.

The most common symptoms in females were headache (17.4%), cough (15.8%) and fever (13.3%). The most commonly reported symptoms among males were cough (12.8%), fever (12.5%) and headache (11.8%). Generally, females exhibited a greater percentage of reported symptoms than males (Figure 2). The distribution of preexisting comorbidities among groups with and without COVID-19-related symptoms, stratified by sex, is shown in Table 2. Males who experienced COVID-19 symptoms were more likely to have asthma than were those who did not experience symptoms (7.2% vs. 3.2%, *p* = 0.002). Compared to those without symptoms, a more significant proportion of female participants who reported symptoms had asthma (10% vs. 7.4%, *p* = 0.04), chronic lung disease (1.5% vs. 0.3%, *p* = 0.004), diabetes mellitus (5.7% vs. 3.6%, *p* = 0.025), chronic kidney disease (1.6% vs. 0.6%, *p* = 0.035) and autoimmune/rheumatic conditions (1.6% vs. 0.5%, *p* = 0.011).

**Table 2:** Distribution of preexisting comorbidities among the study groups with and without COVID-19-related symptoms stratified by sex.

	Male (*n* = 1,241)			Female (*n* = 2,067)		
Comorbidities	With COVID-19 related symptoms (*n* = 347, 27.9%)	Without COVID-19 related symptoms (*n* = 894, 72.0%)	*p* value	With COVID-19 related symptoms (*n* = 680, 32.9%)	Without COVID-19 related symptoms (*n* = 1,387, 67.1%)	*p* value
Asthma	25 (7.2)	29 (3.2)	0.002*	68 (10.0)	102 (7.4)	0.040*
Chronic lung disease	3 (0.9)	2 (0.2)	0.137	10 (1.5)	4 (0.3)	0.004*
Chronic obstructive pulmonary disease	3 (0.9)	3 (0.3)	0.358	6 (0.9)	13 (0.9)	0.902
Hypertension	47 (13.5)	95 (10.6)	0.147	118 (17.4)	210 (15.1)	0.196
Diabetes mellitus	18 (5.2)	47 (5.3)	0.960	39 (5.7)	50 (3.6)	0.025*
Coronary heart disease	2 (0.6)	6 (0.7)	1.000	9 (1.3)	8 (0.6)	0.077
Chronic liver disease	1 (0.3)	2 (0.2)	1.000	5 (0.7)	3 (0.2)	0.124
Chronic kidney disease	4 (1.2)	5 (0.6)	0.276	11 (1.6)	9 (0.6)	0.035*
Autoimmune/Rheumatic condition	2 (0.6)	3 (0.3)	0.623	11 (1.6)	7 (0.5)	0.011*

*Indicates statistical significance (*p* value < 0.05).

The results of the multiple logistic regression analysis, which was conducted separately for males and females, are summarized in Table 3. The Hosmer–Lemeshow test indicated a good model fit. Model 1 indicated that participants with any comorbidity were at a greater risk of experiencing symptoms related to COVID-19 than were those without comorbidities across both sexes (male: OR = 1.53, 95% CI = 1.11–2.12; *p* = 0.01; female: OR = 1.51, 95% CI = 1.21–1.89, *p* < 0.001). For specific comorbidities (Model 2), asthma remained associated with self-reported COVID-19 symptoms among male participants (OR = 2.15, 95% CI = 1.20–3.85; *p* = 0.010). For females, self-reported COVID-19 symptoms were associated with chronic lung disease (OR = 5.39, 95% CI = 1.52–19.18, *p* = 0.009), hypertension (OR = 1.33, 95% CI = 1.00–1.77, *p* = 0.047) and diabetes mellitus (OR = 1.70, 95% CI = 1.07–2.71, *p* = 0.025).

**Table 3:** The association between preexisting comorbidities and self-reported COVID-19-related symptoms stratified by sex.

		Male (*n* = 1,241)			Female (*n* = 2,067)			*p* value for interaction between sex and comorbidity/marital status
Model	Variables	OR	95% CI	*p* value	OR	95% CI	*p* value	
Model 1	Presence of ≥1 comorbidities (Ref = without any comorbidities)	1.53	1.11–2.12	0.010*	1.51	1.21–1.88	<0.001*	0.526
	Marital status (Ref = Single)							0.042*
	Married	1.26	0.91–1.74	0.164	1.37	1.08–1.74	0.01*	
	Divorced and others^a^	0.85	0.62–1.17	0.311	1.34	1.07–1.68	0.011*	
Model fit	Hosmer-Lemeshow test	*p* value = 0.116			*p* value = 0.075			
Model 2	Comorbidities							
	Asthma	2.15	1.20–3.85	0.010*	1.32	0.95–1.85	0.103	0.151
	Chronic lung disease	3.53	0.31–40.37	0.310	5.39	1.52–19.18	0.009*	0.742
	Chronic obstructive pulmonary disease	1.80	0.24–13.72	0.570	0.49	0.14–1.71	0.262	0.267
	Hypertension	1.31	0.87–1.99	0.196	1.33	1.00–1.77	0.047*	0.551
	Diabetes mellitus	0.94	0.51–1.72	0.831	1.70	1.07–2.71	0.025*	0.230
	Coronary heart disease	0.63	0.08–5.26	0.669	1.79	0.60–5.38	0.299	0.416
	Chronic liver disease	0.13	0.00–20.37	0.430	1.58	0.26–9.65	0.618	0.312
	Chronic kidney disease	1.54	0.32–7.40	0.591	1.48	0.55–4.03	0.440	0.888
	Autoimmune/Rheumatic condition	1.71	0.16–18.21	0.658	2.25	0.79–6.45	0.130	0.882
	Marital status (Ref. = Single)							0.051
	Married	1.27	0.92–1.76	0.144	1.35	1.07–1.72	0.013*	
	Divorced and others^a^	0.84	0.61–1.16	0.286	1.33	1.06–1.67	0.014*	
Model fit	Hosmer-Lemeshow test	*p* value = 0.182			*p* value = 0.190			

The analysis was adjusted for age, BMI, smoking status, education level, marital status, race, place of residence, household size, working in medical settings, and vaccinated for COVID-19. ^a^Indicates including free union, separated, and widowed; *Indicates statistical significance (*p* value < 0.05). Ref. represents the reference group.

The interactions between sex and comorbidities were not significant. Further, there was an interaction between sex and marital status in relation to COVID-19 symptoms (p = 0.042 in Model 1 and 0.054 in Model 2, Table 3). In analyses stratified by sex, higher risks of COVID-19 symptoms were found in married females (Model 1 OR: 1.35; 95% CI = 1.07–1.72, *p* = 0.013; Model 2 OR: 1.34; 95% CI = 1.06–1.70, *p* = 0.016), and divorced and others (Model 1 OR: 1.33; 95% CI = 1.06–1.67, *p* = 0.014; Model 2 OR: 1.32; 95% CI = 1.05–1.66, *p* = 0.016) compared to single females. However, no significant association existed between marital status and COVID-19 symptoms in males. After multiple imputations of missing data, the analyses yielded results similar to the primary analyses (Supplementary Table S1).

## 4 Discussion

Among the 3,308 participants who underwent community-based rapid testing in the Dominican Republic, 31% reported having symptoms during the COVID-19 pandemic (between September 2021 and December 2021). Our findings revealed sex differences in COVID-19-related symptom manifestations, preexisting comorbidities, and correlations between particular comorbidities and symptom occurrence. The most commonly reported symptoms across sexes were headache, cough, and fever. These symptoms were similar to those reported to be associated with the delta variant wave, which occurred at the time of our data collection period (25). However, a greater proportion of females than males exhibited symptoms. These findings are consistent with the literature (15, 17), which indicates that the most frequent mild symptoms are cough and headache, with females exhibiting a greater prevalence than males. A possible explanation for this might be the heterogeneity in behavioral factors such as smoking and hormonal influences on the immune response (26).

Our results revealed that the predominant comorbidities observed among participants who underwent rapid COVID-19 testing included asthma, hypertension, diabetes, and chronic lung diseases. This observation is consistent with previous literature (13, 18, 21, 27), which has identified hypertension and diabetes as the most prevalent comorbidities among individuals with COVID-19. We also noticed that asthma was among the top 2 preexisting comorbidities in our study for individuals with an average age of 40 years. Previous studies have shown that among COVID-19 patients, asthma is more prevalent in younger age groups (27, 28). In addition, the higher prevalence of asthma may stem from individuals being more aware of their symptoms when participating in screening, which led them to be included in this study.

Previous reports indicated that the presence of comorbidities was associated with a greater risk for post-COVID-19 symptoms (29) and severe COVID-19 (30). Our findings supported that at least one comorbidity was associated with a greater risk of self-reported COVID-19-related symptoms in both males and females. However, sex differences exist in the specific types of preexisting comorbid conditions that are associated with COVID-19 symptoms. Regarding this observation, at least two aspects should be considered: the sex differences in the prevalence of specific preexisting comorbid conditions and the accessibility to COVID-19 infection.

There is a sex difference in the prevalence of asthma. Asthma is predominantly seen in males before puberty, but after puberty, females have a higher prevalence (31). We found that male participants with asthma were more likely to present with COVID-19-related symptoms compared to females. This finding is similar to previous studies based on sex-aggregation data indicating that asthma is associated with COVID-19 infection (32–34). A review article mentioned that asthma was associated with the infection of COVID-19 (32). Asthma, a chronic respiratory disease, is associated with the perception of the perception regarding the threat of COVID-19 to one’s health (33). In patients currently infected with COVID-19, asthma has been linked to an increased risk of developing severe or critical illness (34). A recent two-sample Mendelian randomization study also revealed that asthma was causally correlated with COVID-19 infection (35). Further study may be needed to examine the potential pathological relationship of the airway mucosal surface with chronic inflammation or lifestyle factors such as smoking.

Sex differences in susceptibility, severity, and progression are widely observed in various chronic respiratory diseases; for example, chronic obstruction pulmonary disease was considered male-predominant (36). In this study, we further found that chronic lung disease was associated with the occurrence of reported symptoms in females. This finding agrees with the literature that revealed that respiratory disease increases the severity of COVID-19 (14, 30).

Diabetes is more common in men at younger ages, but women have more significant risk factors like obesity and stress, contributing to a higher burden at diagnosis (37). We observed that diabetes is associated with an increased risk of COVID-19-related symptoms in females. In line with other studies, diabetic patients were more likely to experience symptomatic COVID-19 infection (13, 38). In a matched case–control study, patients with diabetes had a higher risk perception of the occurrence of COVID-19-related symptoms (39). One possible explanation is the impact of diabetes or genetic susceptibility to the severity of COVID-19 (40).

Sex differences in the incidence and severity of hypertension are well-established, and these differences appear to be age-dependent (41). For example, the prevalence of uncontrolled hypertension was higher in men than women at ages 43 to 46 years but became higher in women than men starting at ages 61 to 64. According to our findings, females with hypertension had a greater likelihood of experiencing symptoms of COVID-19. Previous studies reported that hypertension was correlated with COVID-19 severity (27, 30). ACE2 receptor expression is elevated in individuals with specific comorbidities, such as hypertension and diabetes. As SARS-CoV-2 primarily targets cells via ACE2 receptors, these underlying health issues can exacerbate the severity of COVID-19. However, more studies are required to examine sex differences in the influence of the innate immune system or sex hormones (26).

Most previous studies have examined sex differences in COVID-19 infection, severity, and mortality (15–18), at the same time, less attention has been paid to the relationship between symptoms and comorbidities or whether this relationship varies by sex. Our study improves the understanding of how the association between comorbidities and COVID-19 symptoms differs in males and females, providing insightful information for sex-specific public health and clinical strategies to treat patients with comorbidities. It remains unclear how sex may affect the associations between comorbidities and COVID-19 symptoms. One possible explanation is that males and females differ in genetics, immune response, sex hormones, and socioeconomic and behavioral factors (19, 20); each influences health and disease. Another explanation could be the differences in the severity of certain comorbidity between males and females (20). Also, the social roles of males and females in the Dominican Republic are distinct. The use of healthcare services might vary between males and females, contributing to sex differences in the associations between specific comorbidities and COVID-19 symptoms observed in this study. Further longitudinal or cohort studies are warranted to explore the possible mechanisms underlying the observed sex differences or the causality between specific comorbidities and COVID-19 symptoms.

Our study has several strengths. This was the first study examining sex differences in the associations between preexisting comorbidities and COVID-19-related symptoms among community-dwelling individuals who underwent rapid COVID-19 testing in the Dominican Republic. Our study samples consisted of a large sample of individuals from four regions covering urban and rural areas considered representative of the Dominican Republic. However, this study had some limitations. First, preexisting comorbidities and symptoms were self-reported, which may have introduced recall bias potentially leading to an underestimation or overestimation of their prevalence. Clarification based on medical records may improve credibility. Unfortunately, we could not access the participant’s medical records due to the regulation. Second, this study was a cross-sectional investigation, which cannot explore the causality of preexisting comorbidities and COVID-19-related symptoms. In addition, including of voluntary participants in our study design may have led to the overrepresentation of symptomatic individuals and self-selection bias. Third, we included only individuals who underwent rapid COVID-19 testing and gathered data from individuals who perceived a necessity for quick screening. The rapid IgM/IgG antibody tests performed in this study have limitations in detecting recent infections, particularly in asymptomatic individuals. Indeed, in the real-world setting, asymptomatic subjects usually do not seek rapid testing for COVID-19. Thus, this fact may introduce misclassification bias regarding COVID-19 status and limit the generalizability of our findings. Fourth, this study was conducted in the Dominican Republic, an upper-middle-income country where most of the population identifies as mixed ethnicity. When extrapolating these results to populations with different racial compositions, caution should be taken. Finally, this study did not collect data on certain confounding factors, such as prior COVID-19 infection status, socioeconomics, physical activity, diet, and healthcare access. As a result, our analyses did not consider the influences of these confounders. Thus, our findings should be interpreted with caution.

In conclusion, this large sample of epidemiological surveys in the Dominican Republic revealed sex differences in the associations between specific preexisting comorbidities and COVID-19-related symptoms. The findings suggest that asthma in males, and chronic lung disease, hypertension, and diabetes mellitus in females significantly predict COVID-19-related symptoms. Our results contribute to the understanding of sex-based differences in COVID-19 morbidity, highlighting the need for sex-specific approaches in public health and clinical practices. Thus, our study may provide valuable information for planning healthcare strategies and informing targeted interventions during pandemics. The utilization of sex-disaggregated data will equip clinicians with the ability to formulate sex-specific healthcare decisions.

## Data Availability

The datasets used and analyzed during the current study are not publicly available. Requests to access the datasets should be directed to cjhsieh@mail.tcu.edu.tw.
