# Supplementary material for: Sex differences in the association between preexisting comorbidities and COVID-19-related symptoms during the COVID-19 pandemic in the Dominican Republic
Source: Front Public Health. 2025 Mar 18;13:1536627. doi: 10.3389/fpubh.2025.1536627 (PMC11959077; doi:10.3389/fpubh.2025.1536627)
Supplement: Supplementary file 1 [file Table_1.docx]

Supplementary Table 1. Sensitivity analysis by imputed data of the association between preexisting comorbidities and self-reported COVID-19-related symptoms stratified by sex.

|  |  | Male (n=1,770) | | | Female (n=3,129) | | | *p* value for interaction between sex and comorbidity |
| --- | --- | --- | --- | --- | --- | --- | --- | --- |
| Model | Variables | OR | 95% CI | *p* value | OR | 95% CI | *p* value |  |
| Model 1 | Presence of ≥ 1 comorbidities  (Ref. = without any comorbidities) | 1.51 | 1.12-2.03 | 0.007* | 1.65 | 1.36-2.01 | <0.001* | 0.980 |
| Model 2 | Comorbidities |  |  |  |  |  |  |  |
|  | Asthma | 1.67 | 0.99-2.80 | 0.054 | 1.40 | 1.04-1.89 | 0.026* | 0.538 |
|  | Chronic lung disease | 2.11 | 0.28-15.96 | 0.471 | 3.08 | 1.07-8.89 | 0.037* | 0.678 |
|  | Chronic obstructive pulmonary disease | 4.16 | 0.95-18.24 | 0.059 | 0.91 | 0.36-2.32 | 0.850 | 0.083 |
|  | Hypertension | 1.41 | 0.96-2.07 | 0.079 | 1.42 | 1.11-1.83 | 0.006* | 0.605 |
|  | Diabetes mellitus | 0.84 | 0.47-1.47 | 0.535 | 1.53 | 1.02-2.30 | 0.041* | 0.146 |
|  | Coronary heart disease | 0.33 | 0.04-2.68 | 0.297 | 1.41 | 0.48-4.14 | 0.537 | 0.231 |
|  | Chronic liver disease | 0.10 | 0.00-10.42 | 0.334 | 0.68 | 0.13-3.69 | 0.659 | 0.412 |
|  | Chronic kidney disease | 2.00 | 0.54-7.37 | 0.296 | 1.97 | 0.77-5.04 | 0.159 | 0.922 |
|  | Autoimmune/Rheumatic condition | 3.28 | 0.46-23.69 | 0.238 | 3.48 | 1.30-9.31 | 0.013* | 0.988 |

The analysis was adjusted for age, BMI, smoking status, education level, marital status, race, place of residence, household size, working in medical settings, and vaccinated for COVID-19. *indicates statistical significance (*p* value <0.05). Ref. represents the reference group.
